# Evaluation of subcortical grey matter abnormalities in patients with MRI-negative cortical epilepsy determined through structural and tensor magnetic resonance imaging

**DOI:** 10.1186/1471-2377-14-104

**Published:** 2014-05-14

**Authors:** Syu-Jyun Peng, Tomor Harnod, Jang-Zern Tsai, Ming-Dou Ker, Jun-Chern Chiou, Herming Chiueh, Chung-Yu Wu, Yue-Loong Hsin

**Affiliations:** 1 Department of Electrical Engineering, National Central University, No. 300, Jhongda Rd, Jhongli City 32001, Taoyuan County, Taiwan; 2 Epilepsy Center, Tzu Chi General Hospital, No. 707, Sec. 3, Chung Yang Rd, Hualien City 97002, Taiwan; 3 Biomedical Electronics Translational Research Center, National Chiao Tung University, No. 1001, University Rd, Hsinchu City 30010, Taiwan; 4 Department of Neurology, Chung Shan Medical University and Chung Shan Medical University Hospital, No. 110, Sec. 1, Jianguo N. Rd, South Dist, Taichung City 40201, Taiwan

**Keywords:** Subcortical grey matter, Neocortical epilepsy, Volumetry, Diffusion tensor imaging

## Abstract

**Background:**

Although many studies have found abnormalities in subcortical grey matter (GM) in patients with temporal lobe epilepsy or generalised epilepsies, few studies have examined subcortical GM in focal neocortical seizures. Using structural and tensor magnetic resonance imaging (MRI), we evaluated subcortical GM from patients with extratemporal lobe epilepsy without visible lesion on MRI. Our aims were to determine whether there are structural abnormalities in these patients and to correlate the extent of any observed structural changes with clinical characteristics of disease in these patients.

**Methods:**

Twenty-four people with epilepsy and 29 age-matched normal subjects were imaged with high-resolution structural and diffusion tensor MR scans. The patients were characterised clinically by normal brain MRI scans and seizures that originated in the neocortex and evolved to secondarily generalised convulsions. We first used whole brain voxel-based morphometry (VBM) to detect density changes in subcortical GM. Volumetric data, values of mean diffusivity (MD) and fractional anisotropy (FA) for seven subcortical GM structures (hippocampus, caudate nucleus, putamen, globus pallidus, nucleus accumbens, thalamus and amygdala) were obtained using a model-based segmentation and registration tool. Differences in the volumes and diffusion parameters between patients and controls and correlations with the early onset and progression of epilepsy were estimated.

**Results:**

Reduced volumes and altered diffusion parameters of subcortical GM were universally observed in patients in the subcortical regions studied. In the patient-control group comparison of VBM, the right putamen, bilateral nucleus accumbens and right caudate nucleus of epileptic patients exhibited a significantly decreased density Segregated volumetry and diffusion assessment of subcortical GM showed apparent atrophy of the left caudate nucleus, left amygdala and right putamen; reduced FA values for the bilateral nucleus accumbens; and elevated MD values for the left thalamus, right hippocampus and right globus pallidus A decreased volume of the nucleus accumbens consistently related to an early onset of disease. The duration of disease contributed to the shrinkage of the left thalamus.

**Conclusions:**

Patients with neocortical seizures and secondary generalisation had smaller volumes and microstructural anomalies in subcortical GM regions. Subcortical GM atrophy is relevant to the early onset and progression of epilepsy.

## Background

Recent studies have demonstrated the importance of cortical-subcortical network interactions in seizure generation and propagation [1,2]. Through several magnetic resonance imaging (MRI) acquisition and processing techniques, investigators explore not only the cortex but also subcortical grey matter (GM) abnormalities in epileptic patients. It has been reported that patients with temporal lobe epilepsy (TLE) and idiopathic generalised epilepsy (IGE) have structural alterations in the subcortical nuclei and, more generally, in the thalamus [3-16]. Furthermore, the changes in subcortical GM correlate with the age at seizure onset and the duration of epilepsy [3,4,10,15,16]. A small number of longitudinal studies have shown that recurrent seizures may lead to progressive microstructural alterations [17,18]. However, few neuroimaging studies have addressed the abnormalities in the subcortical GM of patients with neocortical epilepsy. Here, we investigated the subcortical GM of patients with neocortical epilepsy and without any identifiable MRI lesion, compared with age-matched controls. Our patients shared a seizure semiology indicating secondary generalisation. First, we demonstrated density changes in subcortical GM using voxel-based morphometry (VBM). We then correlated the volume changes and diffusion parameters of seven subcortical regions (the hippocampus, caudate nucleus, putamen, globus pallidus, nucleus accumbens, thalamus and amygdala) with age at seizure onset and disease duration. Our aim was to determine the associations between changes in subcortical GM and disease progression, especially in patients whose seizures arise from neocortical structures.

## Methods

### Subjects

From 2012 May to December, we conducted in this neuroimaging study. We studied 24 patients (15 females and 9 males, mean age = 25.6 ± 12.9 years) with chronic partial epilepsy. All patients had had MRI scans and had long-term EEG records. We first selected epileptic patients with regional epileptiform discharges using a data set on patients at the Buddhist Tzu Chi Epilepsy Center. We termed patients “MRI-negative” if radiologists did not identify any lesions, including neoplasms, traumatic lesions, vascular anomalies, well-defined developmental abnormalities or hippocampal atrophy, in their routine brain MRIs. To completely exclude mesial temporal lobe epilepsy, we did not include patients with maximal ictal/interictal epileptiform discharges at T3, T4 or sphenoid electrodes. We also determined the location of the seizure focus or foci in individual patients through ictal video-EEG recording. We termed a focus “undetermined” if seizure activity arose on the EEG in bilateral frontal regions simultaneously or if there was a diffuse epileptiform discharge with asymmetric body posturing at seizure onset. All of the enrolled patients had seizure manifestations with the subsequent development of generalised convulsions and postictal psychomotor depression. Patient demographic information is shown in Table 1. Twenty-nine age-matched healthy volunteers (14 females and 15 males with a mean age of 27.5 ± 4.2 years) were recruited as the control group. The consent in which informed the research methodology and for publication of data and images was obtained from each participant and/or his/her parents. The study protocol was approved by the Research Ethics Committee at Buddhist Tzu Chi General Hospital (IRB 101–32 and IRB101-99).

**Table 1:** Clinical data on 24 patients with focal neocortical epilepsy

**ID**	**Gender**	**Age**	**Age at onset**	**Seizure focus/foci**
1	F	21	12	Undetermined
2	F	42	36	R F, T
3	M	15	14	L F
4	F	25	8	L T
5	M	42	12	R T
6	F	24	2	R O
7	F	16	1	R F, T, O
8	M	30	2	L O
9	M	18	5	R F
10	F	22	6	L T
11	F	11	5	R and L F
12	M	15	3	Undetermined
13	F	31	2	R F
14	F	12	9	Undetermined
15	M	63	10	R F
16	F	14	14	R’t T
17	M	21	Unclear	L O
18	F	40	Unclear	L F
19	F	16	16	Undetermined
20	M	45	31	L T
21	F	32	16	L F
22	M	18	6	R F, T
23	F	25	22	L F
24	F	17	17	R F

F = Frontal; T = Temporal; O = Occipital; Y = Yes; N = No; R = Right hemisphere and L = Left hemisphere; Undetermined = seizure activity arising on the EEG in bilateral frontal regions or diffuse epileptiform discharge with asymmetric body posturing at seizure onset.

### MRI acquisition

All subjects were scanned in a 3T MRI scanner (General Electric, Waukesha, WI, USA). Anatomic T1-weighted images were acquired using a high-resolution, axial, three-dimensional, T1-weighted, fast spoiled gradient recalled echo (3D T1-FSPGR) sequence. Congruent slices with a thickness of 1 mm were generated with a repetition time (TR) of 11.812 ms, an echo time (TE) of 5.036 ms, a field of view (FOV) of 22 × 22 cm, a flip angle of 15 degrees and a 512 × 512 matrix. The DTI protocol consisted of a single-shot-spin-echo planar-imaging sequence. Thirty-four contiguous slices were acquired with a matrix size of 256 × 256, a voxel size of 1 mm × 1 mm, a slice thickness of 3 mm, a TR of 8,000 ms, a TE of 82.4 ms, a number of excitation of 2 and a FOV of 25 × 25 cm. Diffusion-weighted images were acquired in 25 directions (b = 1000 s/mm^2^), as was a null image (b = 0 s/mm^2^).

### VBM analysis of whole brain GM

VBM was carried out using the FSL-VBM v1.1 software tool included in the FSL (FMRIB Software Library; the University of Oxford). The VBM analysis procedure comprised the following steps [19]. First, 3D T1-FSPGR images were brain-extracted and GM -segmented before being registered to the Montreal Neurological Institute (MNI) 152 standard space using non-linear registration. The resulting images were averaged and flipped along the x-axis to create a left-right symmetric, study-specific GM template. Second, all native GM images were non-linearly registered to this study-specific GM template and “modulated” to correct for local expansion (or contraction) due to the non-linear component of the spatial transformation. The modulated GM images were then smoothed with an isotropic Gaussian kernel with a sigma of 3 mm for the TFCE-based analysis [20]. Finally, differences in cerebral GM density between the patient and control groups were evaluated using the voxelwise generalised linear model applied using permutation-based non-parametric testing (5000 permutations) [21]. We identified the regions with significant differences in GM density between the patient and control groups using these postprocessing methods and a cluster-size threshold of *p* < 0.05.

### Measurement of volumes and diffusion parameters of subcortical GM structures

**Figure 1:**
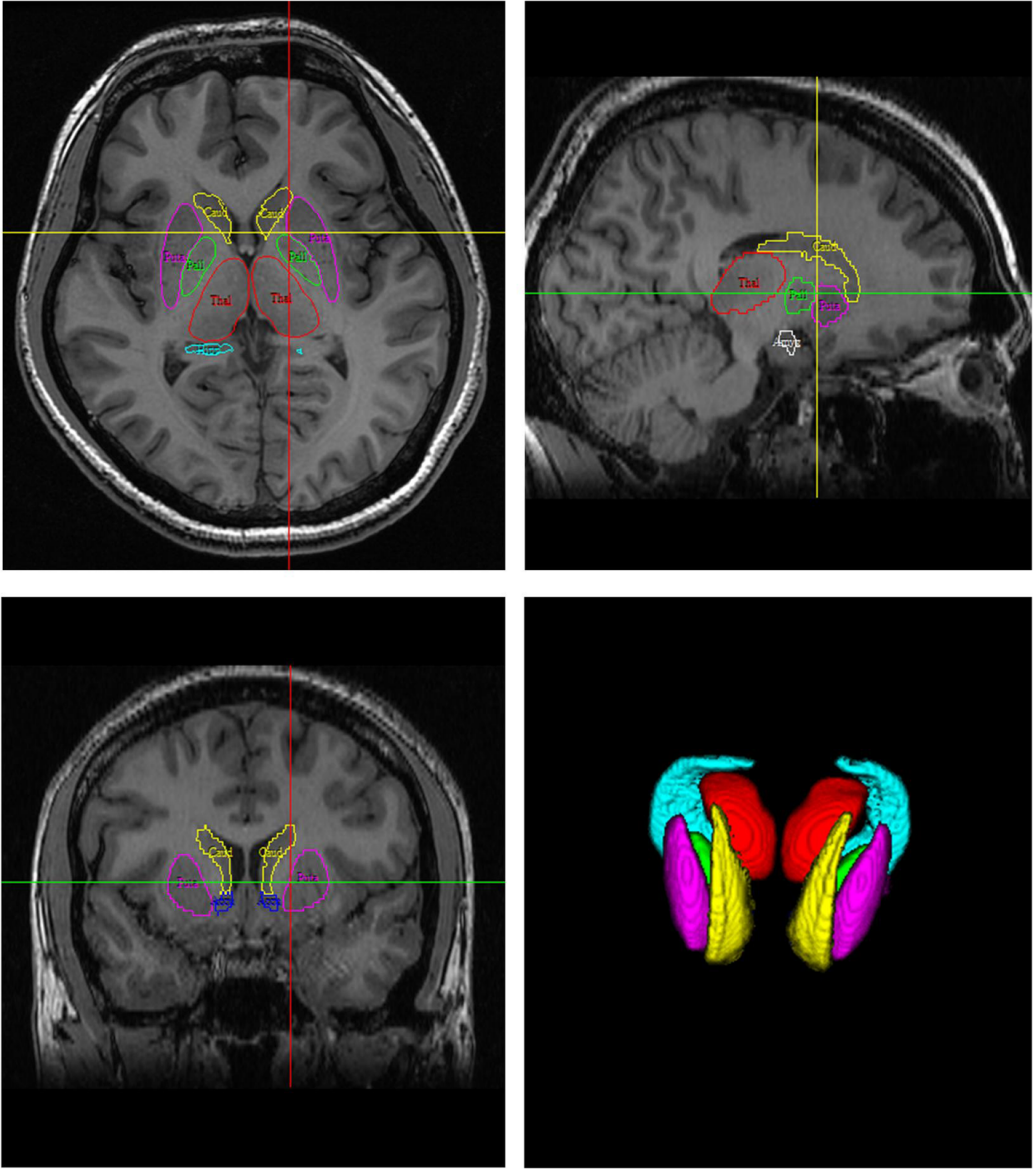
**FIRST segmentation.** Example showing the seven subcortical regions studied (hippocampus, caudate nucleus, putamen, globus pallidus, nucleus accumbens, thalamus and amygdala) in axial, sagittal, coronal and 3D views (hippocampus = cyan; caudate nucleus = yellow; putamen = magenta; globus pallidus = green; nucleus accumbens = blue; thalamus = red; amygdala = white).

The algorithm FIRST (FMRIB’s Integrated Registration and Segmentation Tool) was applied to separately evaluate the left and right volumes of seven subcortical regions: hippocampus, caudate nucleus, putamen, globus pallidus, nucleus accumbens, thalamus and amygdala [22,23]. During registration, the 3D T1-FSPGR images were transformed to the MNI 152 standard space using affine transformations with 12 degrees of freedom. A subcortical mask was applied to locate the different subcortical structures, followed by segmentation based on shape models and voxel intensities after subcortical registration. Finally, a boundary correction was used to determine which boundary voxels belong to a given structure. In this study, a *Z*-value of 3 was used, corresponding to a structure. After the registration and segmentation of all MRI images, all segmented subcortical regions were visually checked for errors in registration and segmentation (Figure 1). The acquired volume of each subcortical structure was normalised to the whole brain volume without cerebrospinal fluid to obtain a volume-ratio value.

All diffusion-weighted images were corrected for eddy current distortion and head motion using the FDT v2.0 software package (FMRIB's Diffusion Toolbox). The preprocessed DTI data were fit to a diffusion tensor model to generate the mean diffusivity (MD) and fractional anisotropy (FA) maps. To obtain transformation parameters, the individual T1-FSPGR image was registered to the null image to fit the DTI resolution using a 12-parameter rigid body transformation. We applied these parameters to transform the segmentation mask to the DTI space using a rigid registration and a nearest neighbour interpolation based on the normalised mutual information method. For each subject, the corresponding values of the MD and FA were calculated for each automatically segmented region.

### Statistical analysis

Using the independent-samples t-test, the normalised volume, FA and MD values in the patient group were compared with those in the control group for the seven subcortical structures studied. To investigate the underlying relation between the significantly altered diffusion parameters or volume of subcortical structures and duration of epilepsy or age at epilepsy onset, linear regression analysis was performed. A significant difference was accepted if the *p* value was less than 0.05.

## Results

We enrolled 24 patients with neocortical epilepsy and without gross cerebral abnormalities. In this study group, more patients had seizures originating in anterior regions of the brain. The proportion of patients with frontal lobe seizures was equal between the right hemispheric epilepsy and left hemispheric epilepsy subgroups.

### VBM analysis

**Figure 2:**
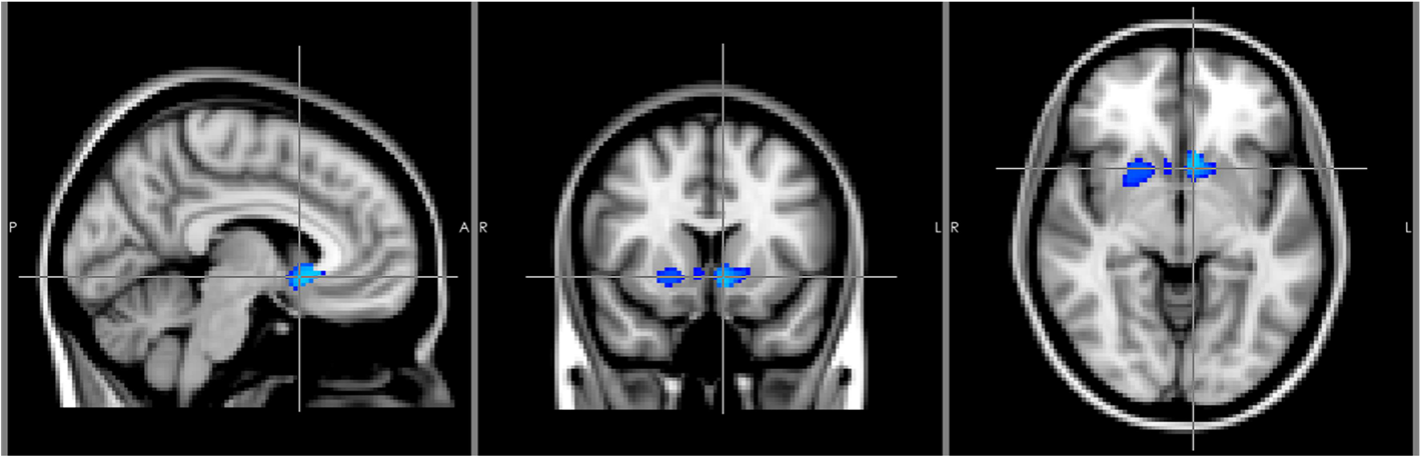
**VBM analysis.** VBM results showing GM volume loss in the bilateral nucleus accumbens, right putamen and right caudate nucleus in neocortical epilepsy patients, compared with controls.

Three clusters exhibited significant decreases in GM density in the whole brain VBM comparison. Within these clusters, the right putamen, the bilateral nucleus accumbens and the right caudate nucleus were involved (Table 2, Figure 2). In addition, an increase in GM density was also observed over the bilateral paracentral gyri in the patient group (Additional file 1: Figure S1).

**Table 2:** **Local maximums of significant clusters showing decreased cerebral GM density in neocortical epilepsy patients, compared to controls (*p*** **< 0.05)**

**Cluster index**	**Anatomy**	**Voxels**	**Z-MAX**	**Z-MAX MNI (mm)**		
				**X**	**Y**	***Z***
1	33% Left Cerebral White Matter	157	0.99	-6	16	-6
	23% Left Nucleus accumbens					
	13% Left Cerebral Cortex					
2	87% Right Putamen	111	0.966	24	10	-8
	12% Right Cerebral White Matter					
3	58% Right Nucleus accumbens	23	0.954	6	12	-4
	19% Right Cerebral White Matter					
	9% Right Caudate nucleus					
	8% Right Cerebral Cortex					
	4% Right Lateral Ventricle					

### Volume difference

The total brain volume was not a confounding factor for the true brain volume (excluding the volume of cerebrospinal fluid), and the true brain volumes of our patients were not different from those of the controls (*t* = 2.009, *p* = 0.615). In general, the studied subcortical structures showed different degrees of volume reduction. The volumes of the left caudate nucleus (2.848 ± 0.469 vs. 3.143 ± 0.506, *t* = 0.430, *p* = 0.034), left amygdala (0.749 ± 0.176 vs. 0.868 ± 0.214, *t* = -0.661, *p* = 0.033) and right putamen (4.002 ± 0.334 vs. 4.297 ± 0.548, *t* = 1.836, *p* = 0.025) were reduced significantly in the patients compared with the controls (Table 3).

**Table 3:** Normalised subcortical structure volumes and FA and MD values in focal neocortical epilepsy patients

**Subcortical structures**	**Volume (×10**^**-3**^**)**				**FA**				**MD (×10**^**-3**^**)**			
	**Controls**	**Patients**	***p***	***t***	**Controls**	**Patients**	***p***	***t***	**Controls**	**Patients**	***p***	***t***
Hipp L	3.011 (0.440)	2.797 (0.547)	0.119	2.052	0.183 (0.019)	0.171 (0.026)	0.052	0.038	1.030 (0.053)	1.074 (0.105)	0.051	-1.870
Caud L	3.143 (0.506)	2.848 (0.469)	0.034*	0.430	0.279 (0.042)	0.275 (0.042)	0.738	-0.336	0.846 (0.069)	0.853 (0.052)	0.669	-2.184
Puta L	4.362 (0.521)	4.128 (0.301)	0.057	1.300	0.194 (0.023)	0.194 (0.024)	0.995	0.006	0.780 (0.031)	0.791 (0.032)	0.200	-1.945
Pall L	1.432 (0.259)	1.438 (0.363)	0.945	1.346	0.353 (0.055)	0.371 (0.069)	0.282	1.086	0.790 (0.036)	0.804 (0.042)	0.184	0.069
Accu L	0.383 (0.116)	0.374 (0.068)	0.723	2.000	0.295 (0.049)	0.265 (0.055)	0.038*	-1.991	0.840 (0.049)	0.832 (0.040)	0.512	-1.585
Thal L	6.300 (0.761)	5.969 (0.454)	0.067	1.608	0.291 (0.026)	0.291 (0.023)	0.970	-0.788	0.863 (0.059)	0.894 (0.050)	0.045*	-2.188
Amyg L	0.868 (0.214)	0.749 (0.176)	0.033*	-0.661	0.197 (0.020)	0.193 (0.018)	0.435	-2.132	0.858 (0.039)	0.877 (0.047)	0.114	-0.357
Hipp R	3.314 (0.427)	3.159 (0.474)	0.216	0.614	0.196 (0.019)	0.188 (0.020)	0.126	1.197	1.042 (0.053)	1.101 (0.102)	0.009*	-1.801
Caud R	3.040 (0.720)	3.032 (0.402)	0.960	1.097	0.260 (0.038)	0.249 (0.038)	0.286	-1.079	0.886 (0.076)	0.913 (0.103)	0.278	-0.051
Puta R	4.297 (0.548)	4.002 (0.334)	0.025*	1.836	0.214 (0.026)	0.206 (0.027)	0.245	-1.176	0.776 (0.034)	0.792 (0.032)	0.072	-2.305
Pall R	1.486 (0.258)	1.428 (0.285)	0.441	2.163	0.403 (0.056)	0.397 (0.058)	0.689	-0.403	0.771 (0.041)	0.979 (0.046)	0.035*	-0.777
Accu R	0.297 (0.109)	0.268 (0.076)	0.275	2.702	0.285 (0.053)	0.240 (0.047)	0.002*	-1.555	0.842 (0.048)	0.847 (0.056)	0.726	-1.253
Thal R	6.086 (0.767)	5.751 (0.538)	0.078	-0.569	0.272 (0.028)	0.281 (0.028)	0.237	-1.715	0.881 (0.062)	0.893 (0.076)	0.542	-1.028
Amyg R	0.788 (0.223)	0.723 (0.237)	0.309	0.353	0.205 (0.020)	0.196 (0.017)	0.092	-3.218	0.869 (0.047)	0.862 (0.043)	0.572	-1.102

Hipp = hippocampus; Caud = caudate nucleus; Puta = putamen; Pall = globus pallidus; Accu = nucleus accumbens; Thal = thalamus; Amyg = amygdala; R = right hemisphere; L = left hemisphere; *denotes a significant difference x*p* < 0.05) with respect to controls.

### Diffusion parameter difference

In general, the MD values for the subcortical structures studied were higher in our patients. The MD value was increased in the left thalamus (0.894 ± 0.050 vs. 0.863 ± 0.059, *t* = -2.188, *p* = 0.045), right globus pallidus (0.979 ± 0.046 vs. 0.771 ± 0.041, *t* = -0.777, *p* = 0.035) and right hippocampus (1.101 ± 0.102 vs. 1.042 ± 0.053, *t* = -1.801, *p* = 0.009). The differences in the FA values were minimal and inconsistent. The FA values were reduced in the bilateral nucleus accumbens in the patients, compared with the controls (left nucleus accumbens, 0.265 ± 0.055 vs. 0.295 ± 0.049, *t* = -1.991, *p* = 0.038; right nucleus accumbens, 0.240 ± 0.047 vs. 0.285 ± 0.053, *t* = -1.555, *p* = 0.002).

### Correlations with age at seizure onset and disease duration

**Figure 3:**
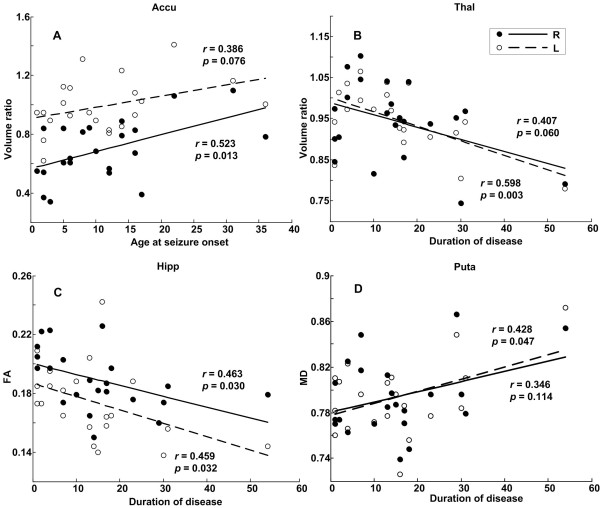
**Clinical correlations of onset age and disease duration. (A)** Linear regressions of the volume ratio of the nucleus accumbens on the age at seizure onset. **(B)** Linear regressions of the volume ratio of the thalamus on disease duration. **(C)** Linear regressions of the FA values of the hippocampus on disease duration. **(D)** Linear regressions of the MD values of the putamen on disease duration. Accu = nucleus accumbens; MD = mean diffusivity; FA = fractional anisotropy; Hipp = hippocampus; Puta = putamen; Thal = thalamus. Volume ratio = the ratio of the individual normalised volume ratio to the mean normalised volume ratio of controls.

The age at seizure onset positively correlated with the volume ratio of the bilateral nucleus accumbens (regression for right nucleus accumbens: *r* = 0.523, *p* = 0.013; regression for left nucleus accumbens: *r* = 0.386, *p* = 0.076) (Figure 3A). The disease duration significantly negatively correlated with the volume ratio of the left thalamus (*r* = 0.598, *p* = 0.003) (Figure 3B), the mean FA of the bilateral hippocampus (left: *r* = 0.459, *p* = 0.032; right: *r* = 0.463, *p* = 0.030) (Figure 3C) and the mean FA of the left putamen (*r* = 0.435, *p* = 0.043). The clinical-MD correlations with epilepsy duration showed a positive trend, but only that for the left putamen reached statistical significance (*r* = 0.428, *p* = 0.047) (Figure 3D). The Additional file 2: Table S1 shows the linear regression relations among normalised volume, the DTI parameters of the subcortical structures and either age at seizure onset or disease duration. The diffusion parameters did not show significant correlations with age at seizure onset.

## Discussion

Generalised tonic-clonic seizures occur in primary generalised epilepsy and can arise as a secondary generalisation of partial seizures. Over 70% of patients with focal seizures experience secondary generalisation [24].

Many studies emphasise the importance of the thalamus in generalised seizures. It has also been demonstrated that the basal ganglia contribute to seizure regulation and ictal dystonia [25,26]. In this study, we have observed that focal cortical seizures with secondarily generalised tonic-clonic convulsions are associated with variable changes in the subcortical GM of individual patients. Subcortical GM atrophy was related to the early onset and progression of epilepsy. Involvement of the nucleus accumbens in secondary seizure generalisation has not been reported previously in humans.

DeCarli first discussed volume asymmetry in the extratemporal structures of patients with complex partial seizures of left temporal origin. In addition to changes in the hippocampus, they also observed the significantly reduced volumes of the left thalamus, left caudate nucleus and bilateral lenticular nuclei [27]. Consequently, the amygdala [15], putamen [10,12-14,16], caudate nucleus [11,14-16], globus pallidus [11] and hippocampus [11,13-15] were also found to show atrophy in patients with temporal lobe epilepsy with or without MRI-visible hippocampal lesions. Thereafter, patients with IGEs, including absence epilepsy, juvenile myoclonic epilepsy (JME) and primary generalised tonic-clonic seizures, were also found to have subcortical abnormalities [3-10]. Here, we further demonstrated that the reduction in volume of subcortical GM in patients with frontal, lateral temporal or occipital lobe seizures is universal. Recently, several studies have addressed differences in the shapes of subcortical structures between patients with generalised epilepsies and normal controls using FSL-FIRST, a vertex-based shape analysis method. Du et al. found significant regional atrophy in the left thalamus, left putamen and bilateral globus pallidus in patients with GTCs [5]. Kim identified regional bilateral atrophy on the anterior-medial and posterior-dorsal aspects of the thalamus in 50 adult patients with IGE [28]. In patients with JME, Saini observed focal surface area reductions in the medial and lateral aspects of the bilateral thalami [3].

For tensor imaging supporting the evaluation of white matter rather than GM, we calculated diffusion parameters for original subcortical GM structures individually instead of by whole brain voxel-based analysis to guard against the possibility of causing partial volume averaging effects via smoothing. Although we did not anticipate a demonstration of the delicate microstructural changes of subcortical GM by DTI, we nonetheless observed a general alteration of diffusion parameters. Furthermore, the decrease in FA values in the bilateral nucleus accumbens has not yet been reported. Groppa reported increases in the regional FA in the thalamus in patients with IGE [9]. Luo and Yang found increased MD values in the bilateral thalami, putamen and left caudate nucleus and increased FA values in the bilateral caudate nuclei in patients with absence epilepsy [4,8]. Keller reported the first evidence of combined microstructural and macrostructural putamen abnormalities in patients with JME and identified an early age at onset and a longer duration of epilepsy as predictors for greater architectural alterations [10]. In patients with TLE and abnormal hippocampal MRI scans, Kimiwada showed an increasing trend in the MD values for the thalami ipsilateral to the epileptic focus, and Keller showed changes in the mean FA values of the bilateral thalamus and putamen [12,29]. In Keller’s study, the duration of epilepsy was significantly negatively correlated with the mean FA of both the ipsilateral thalamus and the contralateral thalamus [12].

Saini found a correlation between age at onset and the volume of the right hippocampus in 40 patients with JME [3]. While the ipsilateral-to-contralateral volume ratios of subcortical structures were estimated using data from 40 patients with TLE, thalamic volume loss was found to correlate with epilepsy onset [15]. In two early studies by Dreifuss and Gärtner, the relations between age at onset or epilepsy duration and volume changes in the thalamus or striatum in patients with temporal lobe epilepsy and extratemporal lobe epilepsy were not significant. However, these two studies included patients with neoplasms or cortical dysplasia, which reflect different temporal lobe epileptogenic processes [16,30]. Luo found significant correlations between diffusion parameters for the caudate nucleus and age at onset in patients with absence seizures [4]. In our patients, the age at seizure onset positively correlated with the volume of the right nucleus accumbens, and the reduction in volume observed with disease progression was consistent across the subcortical structures studied, especially the left thalamus.

In 2010, Hermann et al. characterised neurodevelopmental changes in brain structure in children with negative MRI scans and new-onset generalised and localisation-related epilepsies (including extratemporal lobe epilepsy). In their prospective study, they observed reductions in the volume of cerebral GM and a delayed age-appropriate increase in white matter volume over 2 years [17]. In 2011, they further concluded that the baseline grey and white matter volumes differed in the controls, suggesting that anomalies in brain development were antecedent to the onset of seizures and that the neurodevelopmental changes that they observed involved several subcortical structures [18]. The results of our cross-sectional study, demonstrating a correlation between structural abnormalities and age at seizure onset or disease duration, are consistent with the results of their prospective study.

With regard to the postictal state, functional brain imaging has been used in a limited number of studies. Fong et al. conducted a single-photon emission computed tomography (SPECT) study of 2 patients with right TLE in whom postictal psychosis developed; these authors reported a marked hyperperfusion of the left basal ganglia [31]. Blumenfeld and his colleague also used SPECT to observe the involvement of the caudate nucleus during seizure generalisation and in the postictal period [32]. The nucleus accumbens, a region of the brain in the basal forebrain, plays a central role in the reward circuit and the in pathogenesis of psychiatric disorders [33,34]. Studying the involvement of the nucleus accumbens in epilepsy models focuses on postictal behaviors [35,36]. Ma et al. found that the μ opioid receptors of nucleus accumbens mediate immediate postictal decrease in locomotion after an amygdaloid kindled seizure in rats [37]. Using the pilocarpine model, Scholl et al. observed neuronal degeneration in the outside hippocampal regions including the nucleus accombens and the accumbens shell. These findings support our findings indirectly and encourage future research the association of nucleus accumbens with epilepsy.

## Conclusions

Subcortical GM involvement in the pathogenesis of chronic neocortical epilepsy is supported by our DTI-derived and T1-weighted MRI-derived evidence. However, a longitudinal study is needed to determine whether neurodegeneration observed in subcortical regions in neocortical epilepsy patients is accelerated beyond the effects of normal aging. Comorbid interictal and postictal psychomotor symptoms also require further investigation in light of coexisting subcortical structural changes.

## Abbreviations

MD: Mean diffusivity; DTI: Diffusion tensor imaging; FA: Fractional anisotropy; FDT: FMRIBs diffusion toolbox; FIRST: FMRIB’s integrated registration and segmentation tool; FOV: Field of view; FSL: FMRIB software library; GM: Grey matter; IGE: Idiopathic generalised epilepsies; JME: Juvenile myoclonic epilepsy; MNI: Montreal neurological institute; MRI: Magnetic resonance imaging; SPECT: Single-photon emission computed tomography; TE: Echo time; TLE: Temporal lobe epilepsy; TR: Repetition time; VBM: Voxel-based morphometry; 3D T1-FSPGR: Three-dimensional, T1-weighted, fast spoiled gradient recalled echo.

## Competing interests

The authors declare that they have no competing interests.

## Authors’ contributions

SJP: draft of manuscript, including description of study, results and analysis. TH: clinical evaluation and study concept. JZT: revision of manuscript for content. MDK, JCC, HC and CYW: interpretation of data and obtaining funding. YLH: study design and supervision. All authors read and approved the final manuscript.

## Pre-publication history

The pre-publication history for this paper can be accessed here:

http://www.biomedcentral.com/1471-2377/14/104/prepub

## Supplementary Material

Additional file 1: Figure S1 FSL-VBM results comparing neocortical epilepsy patients with controls indicate a bilateral elevation in GM volume over the paracentral gyri in subjects with neocortical epilepsy. Click here for file

Additional file 2: Table S1 Results of the linear regression analysis of normalised volume, DTI parameters of the subcortical structures and either age at seizure onset or disease duration. Click here for file
