# Supplementary figures and images for: Evaluation of subcortical grey matter abnormalities in patients with MRI-negative cortical epilepsy determined through structural and tensor magnetic resonance imaging
Source: BMC Neurol. 2014 May 14;14:104. doi: 10.1186/1471-2377-14-104 (PMC4080585; doi:10.1186/1471-2377-14-104)

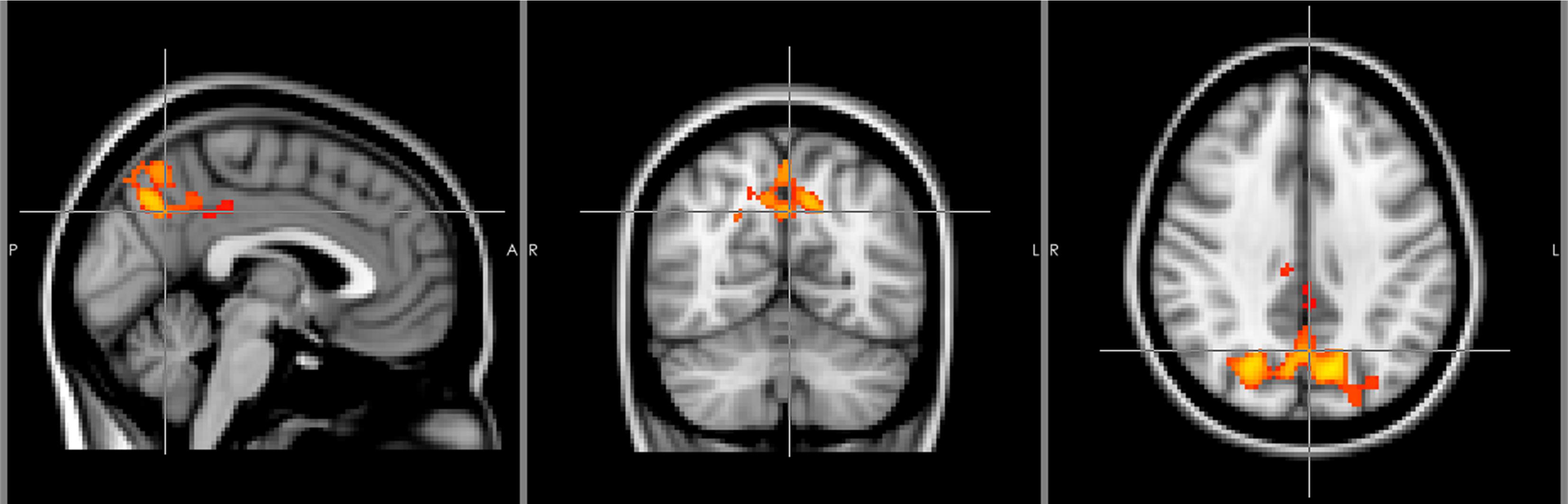

Supplement: Additional file 1: Figure S1 — FSL-VBM results comparing neocortical epilepsy patients with controls indicate a bilateral elevation in GM volume over the paracentral gyri in subjects with neocortical epilepsy. [file 1471-2377-14-104-S1.jpeg]
