# Supplementary material for: Evaluation of subcortical grey matter abnormalities in patients with MRI-negative cortical epilepsy determined through structural and tensor magnetic resonance imaging
Source: BMC Neurol. 2014 May 14;14:104. doi: 10.1186/1471-2377-14-104 (PMC4080585; doi:10.1186/1471-2377-14-104)
Supplement: Additional file 2: Table S1 — Results of the linear regression analysis of normalised volume, DTI parameters of the subcortical structures and either age at seizure onset or disease duration. [file 1471-2377-14-104-S2.docx]

Additional file 2: Table S1 Results of the linear regression analysis of normalised volume, DTI parameters of the subcortical structures and either age at seizure onset or disease duration

| Subcortical  structures |  | Volume vs. age  at seizure onset | |  | FA vs. age  at seizure onset | |  | MD vs. age  at seizure onset | |  | Volume vs.  disease duration | |  | FA vs.  disease duration | |  | MD vs.  disease duration | |
| --- | --- | --- | --- | --- | --- | --- | --- | --- | --- | --- | --- | --- | --- | --- | --- | --- | --- | --- |
|  |  | *r* | *p* |  | *r* | *p* |  | *r* | *p* |  | *r* | *p* |  | *r* | *p* |  | *r* | *p* |
| Hipp L |  | 0.133 | 0.556 |  | 0.200 | 0.372 |  | 0.164 | 0.465 |  | 0.247 | 0.267 |  | 0.459 | 0.032* |  | 0.354 | 0.106 |
| Caud L |  | 0.012 | 0.957 |  | 0.060 | 0.792 |  | 0.235 | 0.292 |  | 0.044 | 0.846 |  | 0.241 | 0.280 |  | 0.067 | 0.768 |
| Puta L |  | 0.037 | 0.870 |  | 0.054 | 0.810 |  | 0.062 | 0.783 |  | 0.052 | 0.820 |  | 0.435 | 0.043* |  | 0.428 | 0.047* |
| Pall L |  | 0.300 | 0.175 |  | 0.152 | 0.499 |  | 0.071 | 0.752 |  | 0.145 | 0.521 |  | 0.323 | 0.143 |  | 0.247 | 0.267 |
| Accu L |  | 0.386 | 0.076 |  | 0.218 | 0.331 |  | 0.171 | 0.447 |  | 0.404 | 0.062 |  | 0.137 | 0.542 |  | 0.036 | 0.874 |
| Thal L |  | 0.072 | 0.752 |  | 0.117 | 0.605 |  | 0.175 | 0.437 |  | 0.598 | 0.003* |  | 0.179 | 0.424 |  | 0.391 | 0.072 |
| Amyg L |  | 0.062 | 0.783 |  | 0.028 | 0.901 |  | 0.198 | 0.376 |  | 0.267 | 0.230 |  | 0.130 | 0.564 |  | 0.085 | 0.706 |
| Hipp R |  | 0.158 | 0.484 |  | 0.279 | 0.209 |  | 0.223 | 0.319 |  | 0.115 | 0.609 |  | 0.463 | 0.030* |  | 0.371 | 0.089 |
| Caud R |  | 0.249 | 0.263 |  | 0.027 | 0.904 |  | 0.019 | 0.934 |  | 0.032 | 0.889 |  | 0.154 | 0.494 |  | 0.276 | 0.215 |
| Puta R |  | 0.293 | 0.185 |  | 0.137 | 0.542 |  | 0.029 | 0.897 |  | 0.149 | 0.508 |  | 0.324 | 0.142 |  | 0.346 | 0.114 |
| Pall R |  | 0.070 | 0.757 |  | 0.216 | 0.333 |  | 0.094 | 0.677 |  | 0.178 | 0.429 |  | 0.155 | 0.492 |  | 0.130 | 0.564 |
| Accu R |  | 0.523 | 0.013* |  | 0.004 | 0.986 |  | 0.021 | 0.927 |  | 0.195 | 0.383 |  | 0.185 | 0.409 |  | 0.000 | 1.000 |
| Thal R |  | 0.167 | 0.458 |  | 0.103 | 0.647 |  | 0.317 | 0.151 |  | 0.407 | 0.060 |  | 0.267 | 0.230 |  | 0.285 | 0.198 |
| Amyg R |  | 0.005 | 0.983 |  | 0.066 | 0.771 |  | 0.179 | 0.424 |  | 0.204 | 0.362 |  | 0.314 | 0.155 |  | 0.179 | 0.425 |

Hipp = hippocampus; Caud = caudate nucleus; Puta = putamen; Pall = globus pallidus; Accu = nucleus accumbens; Thal = thalamus; Amyg = amygdala; R = right; L = left; * denotes a significant difference, with *p* < 0.05.
